# Identification of QTL for Early Vigor and Stay-Green Conferring Tolerance to Drought in Two Connected Advanced Backcross Populations in Tropical Maize (*Zea mays* L.)

**DOI:** 10.1371/journal.pone.0149636

**Published:** 2016-03-21

**Authors:** Samuel Trachsel, Dapeng Sun, Felix M. SanVicente, Hongjian Zheng, Gary N. Atlin, Edgar Antonio Suarez, Raman Babu, Xuecai Zhang

**Affiliations:** 1 The International Maize and Wheat Improvement Center (CIMMYT), International Apdo. Postal 6–641, 06600 Mexico, D.F. Mexico; 2 CIMMYT, ICRISAT Campus, Patancheru, Hyderabad, 502 324, India; 3 Shanghai Academy of Agricultural Sciences (SAAS), Postal 201106, Shanghai, China; Huazhong university of Science and Technology, CHINA

## Abstract

We aimed to identify quantitative trait loci (QTL) for secondary traits related to grain yield (GY) in two BC_1_F_2:3_ backcross populations (LPSpop and DTPpop) under well-watered (4 environments; WW) and drought stressed (6; DS) conditions to facilitate breeding efforts towards drought tolerant maize. GY reached 5.6 and 5.8 t/ha under WW in the LPSpop and the DTPpop, respectively. Under DS, grain yield was reduced by 65% (LPSpop) to 59% (DTPpop) relative to WW. GY was strongly associated with the normalized vegetative index (NDVI; r ranging from 0.61 to 0.96) across environmental conditions and with an early flowering under drought stressed conditions (r ranging from -0.18 to -0.25) indicative of the importance of early vigor and drought escape for GY. Out of the 105 detected QTL, 53 were overdominant indicative of strong heterosis. For 14 out of 18 detected vigor QTL, as well as for eight flowering time QTL the trait increasing allele was derived from CML491. Collocations of early vigor QTL with QTL for stay green (bin 2.02, WW, LPSpop; 2.07, DS, DTPpop), the number of ears per plant (bins 2.02, 2.05, WW, LPSpop; 5.02, DS, LPSpop) and GY (bin 2.07, WW, DTPpop; 5.04, WW, LPSpop), reinforce the importance of the observed correlations. LOD scores for early vigor QTL in these bins ranged from 2.2 to 11.25 explaining 4.6 (additivity: +0.28) to 19.9% (additivity: +0.49) of the observed phenotypic variance. A strong flowering QTL was detected in bin 2.06 across populations and environmental conditions explaining 26–31.3% of the observed phenotypic variation (LOD: 13–17; additivity: 0.1–0.6d). Improving drought tolerance while at the same time maintaining yield potential could be achieved by combining alleles conferring early vigor from the recurrent parent with alleles advancing flowering from the donor. Additionally bin 8.06 (DTPpop) harbored a QTL for GY under WW (additivity: 0.27 t/ha) and DS (additivity: 0.58 t/ha). R^2^ ranged from 0 (DTPpop, WW) to 26.54% (LPSpop, DS) for NDVI, 18.6 (LPSpop, WW) to 42.45% (LPSpop, DS) for anthesis and from 0 (DTPpop, DS) to 24.83% (LPSpop, WW) for GY. Lines out-yielding the best check by 32.5% (DTPpop, WW) to 60% (DTPpop, DS) for all population-by-irrigation treatment combination (except LPSpop, WW) identified are immediately available for the use by breeders.

## Introduction

On a global scale more than 800 million people suffer from energy and/or protein deficiencies. As maize accounts for a high percentage of total cereal production, the successful and continuous production of maize is key to global food security [1]. Drought stress is one of the most important abiotic constraints to agricultural production. In conjunction with high temperatures, drought will have even stronger negative effects on agricultural production in coming decades as a result of climate change. It has been projected that by the year 2050, a 70% increase in global food production must occur, while the global climate change scenario tends to increase the problems of food insecurity [2]. Each degree day above 30°C reduced the final yield by 1% under optimal rain-fed conditions, and by 1.7% under drought conditions [3]. Roughly 65% of present maize-growing areas in Africa would experience yield losses per 1°C of warming under optimal rain-fed management, with 100% of areas harmed by warming under drought conditions.

In order to cope with anticipated climate change it is indispensable to develop drought tolerant maize. To cope with dry environmental conditions plants have developed different strategies [4]: escape and resistance. While escape is related to the completion of the life cycle before the onset of severe stress, resistance is related to dehydration avoidance and tolerance. In practice escape to drought stress is achieved by selection for an earlier flowering date, most likely incurring a yield penalty under optimum conditions. Plants show greatest susceptibility to drought stress during flowering as reproductive processes are affected by water deficiency and drought induced assimilate scarcity: Drought stress around flowering will reduce silk elongation rate [5], increase the anthesis silking interval [6] and result in reduced ovule fertilization and increased kernel abortion. Flowering before the onset of growth limiting drought is an important way to ensure yield formation in environmental conditions with insecure rainfall/ terminal drought [7]. In the past selection for drought tolerance has been carried out for improved harvest index, stay green and shorter ASI [8]. Germplasm was evaluated in multilocation trials for its performance under optimum conditions and only at the very last stage under drought stress before release. Although selection effectiveness for drought tolerance has been improved under managed stress [9], it remains fairly inefficient relative to selection under optimum conditions as yield drops, genetic variance is reduced and genotype-by-environment interaction is poorly understood [10, 11]. Under these conditions, secondary traits may increase selection efficiency, provided they have high adaptive value, strong genetic correlation with GY, high heritability and are easy to measure [12]. It has been demonstrated that some secondary traits, such as the anthesis-silking interval (ASI), ears per plant (EPP), plant height (PHT) and stay-green traits (leaf senescence and chlorophyll content), are correlated with drought response and remain stable under drought stress or might even exhibit enhanced genetic variance [8, 13, 14, 15]. These traits were extensively used in selection at CIMMYT over the past decades reducing genetic variance [16] in the case of ASI.

In order to maintain grain yield and selection gains high in the future it is necessary to identify new secondary traits associated with grain yield. A desirable secondary trait should be genetically correlated with grain yield, exhibit adequate genetic variability, record moderate to high heritability, easy and economical to measure in the field, lend itself for reliable assessments with individual plants or in small plots and have no yield penalty in non-stressed environments [16, 17]. Two such new secondary traits are early vigor measured as normalized differential vegetative index (NDVI) and stay green scored visually. Canopy senescence in maize begins before the leaf area is fully developed, and continues at an increased rate during the grain-filling period [18]. It is generally used by breeders as an indicator to approximate a crops’ phenological/ physiological status after flowering as it correlates with physiological maturity [19].

Early vigor is especially important since it was shown [19] that within a 2 week maturity group early flowering hybrids could yield as much as later flowering hybrids if selection for earlier flowering was carried out in conjunction with good early vigor and stay green. Early vigor can be estimated in a fast, non-destructive manner using spectroradiometric techniques. The NDVI measured with a spectroradiometer can be used to quantify the development of biomass over time [20]. Most traits (including NDVI and senescence) can only be measured reliably when testcrosses are evaluated since the correlation between lines and testcrosses is rather low. The identification of molecular markers associated with grain yield and secondary traits would allow selecting for the presence/absence of markers in nurseries allowing targeted selection at a very early stage. It would furthermore contribute to the understanding of the genetic basis of crop performance especially under drought stressed conditions allowing designing cost-effective breeding approaches aimed at improving grain yield under adverse conditions [21].

Marker assisted selection using QTL has the potential to increase the efficiency of traditional breeding strategies for improvement of varieties [22]. The response of plants to drought stress is of polygenic inheritance and involves expression of many genes and pathways for diverse mechanisms and interactions with environments. Drought tolerance QTL studies in maize and other crops and the strategies for their use in marker-assisted selection in breeding programs have been extensively discussed in several comprehensive reviews [17, 21, 22, 23, 24, 25]. To date a multitude of QTL have been found for grain yield under well-watered [14, 15, 26] and drought stressed conditions [26, 27, 28] for secondary traits such as general vigor [29], stay green [27, 28] and even root traits [30, 31, 32].

As a result of cost and technical feasibility many studies carried out in the past 5 years have rarely used more than several hundred markers molecular markers (SNPs). As a result the confidence interval surrounding a QTL is usually large, making it difficult to precisely locate a QTLs position or use in marker assisted selection. New technologies like next generation sequencing have made the fast, low cost and reliable identification of thousands of molecular markers possible. A next-generation molecular marker discovery approach called genotyping by sequencing (GBS), has been proposed to increase the availability of thousands of SNP molecular markers evenly distributed throughout the genome [33, 34]. Using GBS will allow the development of genetic maps with a high resolution and the precise mapping of QTL.

Many studies carried out in the past have limited value for breeding for various reasons: Evaluation of recombinant inbred lines (RILs; F7 single seed descent lines) with little predictive value of testcross performance; use of a limited number of populations not allowing the validation of QTL across genetic backgrounds; use of marker densities too low to map QTL with a sufficiently high genetic resolution; strong focus on only grain yield under drought, thereby neglecting secondary traits. In the current study, we propose to address all of these points. We used two BC_1_F_2:3_ backcross QTL populations (ABQTL), which were developed by crossing an elite line (CML491) with two advanced donors for drought tolerance [35], testcrossed to CML503 for the phenotypic evaluation. Using such a population structure on the one hand allows mapping QTL; On the other hand it would allow the introgression and pyramidization of genetic segments increasing tolerance to drought from either donor (LPSC7F64; DTPWC9F104) into an elite line (CML491) using marker assisted backcross selection in a very short time [36]. The use of secondary traits (namely NDVI and senescence) in addition to grain yield, will allow to compensate for the reduced genetic variance in ‘old secondary traits’, such as the ASI [16], enabling us to maintain or increase selection efficiency in the future. The use of genotyping by sequencing will allow us to obtain a genetic map with a high resolution (several 1000 SNPs) at reduced cost (~20–35 USD/ entry) enabling us to precisely map QTL for grain yield and secondary traits.

The objectives of the current study were to identify i) secondary traits associated with grain yield; ii) QTL for secondary traits and grain yield in two related BC_1_F_2:3_ populations under well-watered and drought stressed conditions.

## Materials and Methods

All trials of this study were carried out in agreement with landowners (CIMMYT, NOVASEM, INIFAP) owning the land used for these trials. Crop management (agronomy) did not have any adverse effects on the natural environment. Crop management treatment (well-watered vs drought stressed) did not have any adverse effect on land outside the trial area.

### Plant material

Two connected BC_1_F_2:3_ populations, LPSpop and DTPpop, were developed with two drought tolerant lines DTPWC9-F104-5-4-1-1-B-B (DTPWC9F104) and La Posta Sequia C7-F64-2-6-2-1-B-B (LPSC7F64), respectively. Inbred line DTPWC9F104 was derived from Family 104 of the Drought Tolerant Population White (DTPW) Cycle 9, a tropical population developed at CIMMYT. DTPW consists of germplasm coming from Corn Belt experimental hybrids and unimproved landraces which performed well under drought conditions at altitudes below 1800 meters. The first step was the formation of DTP1 which consisted of 14 drought tolerant sources. Four cycles of half sib recurrent selection were made under drought stressed conditions. The DTP1C5 population was crossed with 25 other drought-tolerant sources to form DTP2. From cycle 0 to 3, improvement was carried out using half sib recurrent selection without drought performance evaluation, from cycle 4 to 9, using full sib recurrent selection under drought stressed conditions. Selection for white kernel was made in cycles 8 and 9 [16]. Different inbred lines like DTPWC9F104 were extracted.

The second inbred line donor, LPSC7F64, is traced to the La Posta Sequia Population. The La Posta Sequia Population is a white dent, Tuxpeño-related synthetic, well adapted to the lowland tropics. Full sib recurrent selection was carried out under drought conditions from cycle 0 to cycle 7.

The recurrent parent CML491, is an elite inbred line which is used as male in composition of three way hybrids released by CIMMYT. CML491 is a late tropical white, quality protein maize [37], dent inbred line belonging to heterotic group A (http://apps.cimmyt.org). 175 and 220 BC_1_F_2:3_families were developed from DTPWC9F104 and LPSC7F64, respectively.

CML503 was used as the tester. CML503 is a lowland tropical, late/intermediate, white dent inbred line belonging to heterotic group B which has a good specific combining ability with CML491 (http://apps.cimmyt.org). Hybrids CML312/CML444, SC-Malawi/CML444 and SC-Malawi/H16 were included in both trials as reference hybrids. CML444 and H16 are lines widely used in hybrids deployed in drought prone areas in sub-Saharan Africa.

### Experimental design and environmental conditions

Ten field experiments (four under well-watered, six under drought stressed conditions), were conducted in Mexico during the winter cycle of 2012, 2013 and 2014. Experiments were carried out in Autlan (Jalisco, Mexico 19.76°N, 104,33°W, 924 masl; AUT), Iguala (Guerrero, Mexico, 18.34°N, 99.50°W, 732 masl; IG), Obregon (Sonora, Mexico, 27.29°N, 109.56°W; 62 masl; OB), Tlatizapan (Morelos, Mexico, 18.69°N, 99.13°W, 940 masl; TL) and Agua Fria (Puebla, Mexico, 20°27'N 97°38'W; 110 masl; AF).

For each environment, the experimental design was an alpha-lattice (0, 1) replicated twice in each environment at a block size of 5. Plots were 4.5 m long and 1 row wide at a row spacing of 0.75 m and an interplant spacing of 0.2 m resulting in a planting density of 6.66 plants/m^2^. Plots were hand-planted with two seeds per hill and thinned to one plant per hill three weeks after planting. Trials were drip-irrigated. Fertilizers, insecticides and herbicides were applied as needed.

Drought stress was induced in TL13A, IG13A, AUT13A, OB13A, IG14A and TL14A. Water deficit was induced by withholding irrigation two weeks (~190 GDD) before flowering. An additional irrigation of 20 l/ m^2^ was applied five days after the mean male flowering date of each trial to ensure proper grain filling. Four environments TL12A, IG14A, TL14A and AF14A were used as well-watered environments during which evapotranspirated water was fully replaced through weekly irrigations. Soil moisture content was measured at 10, 20, 30, 40, 60 and 100 cm soil depth three times weekly using Delta-t PR2/6 soil moisture probes (Delta-T devices, Cambridge, United Kingdom) to schedule irrigations in the drought stress treatment.

### Phenotypic data acquisition

A set of traits have been measured for each plot, throughout the cropping cycle at different stages of development. Two, four, five and six weeks after planting the normalized differential vegetative index (NDVI) was measured using an RT-505 Greenseeker (Trimble, Ukia, CA, USA). NDVI measurements were taken by running the sensor in the middle of each plot at a height of 80 cm above the canopy. NDVI was calculated according to the following equation:

$$NDVI\;=\;\frac{R_{NIR}-R_{Red}}{R_{NIR}+\;R_{Red}}$$

where *R*_*NIR*_ is the reflectance of near infrared wavelength, and *R*_*Red*_ is the reflectance of red wavelength. NDVI illustrates the part of red wavelength which is absorbed by the plant.

At flowering, anthesis (AD) and silking date (SD) were recorded when 50% of plants within a plot were shedding pollen, and growing silks respectively. The anthesis silking interval (ASI) was calculated as the difference between male and female flowering. Four and six weeks after flowering senescence was measured visually using a scale ranging from 1 (no senescence) to 9 (complete senescence) to approximate stay green. The area under the curve (AUC) for NDVI and senescence was calculated by integrating a polynomial function of second degree fitted to individual measurements taken before (for NDVI) or after flowering (for senescence). Grain yield (GY) was calculated based on dry shelled grain yield and is reported at 12% moisture.

### Phenotypic data analysis

The mixed effect linear model used for the analysis of phenotypic data measured in multilocation trials was:

$$y_{mhlk}=\mu +a_{h}+E_{ml}+a_{h}E_{ml}+r\left(E_{ml}\right)+r\left(E_{ml}\right)\delta_{k}+\varepsilon_{mhlk}$$

Where Y_*hmlk*_ is the trait value of the h^th^ genotype (LPSpop: h = 220; DTPpop: h = 175) for the l^th^ environment (WW: l = 4; DS: l = 6), the m^th^ replication (m = 2); *μ* the overall mean, *a*_*h*_ the main effect of the genotype, *E*_*ml*_ the effect of the environment, *a*_*h*_*E*_*ml*_ the genotype-by-environment interaction, *r*(*E*_*ml*_) the replication within environment effect and *r*(*E*_*ml*_)*δ*_*k*_ the effect of blocks within replicates within environments and the random error term ε_*mhlk*_. All factors were set as random factors. Best linear unbiased predictors (BLUP) of genotypes, variance components, and broad sense heritability were obtained. Since physiological mechanisms conveying grain yield are expected to be different under well-watered and drought stressed treatments, data for both irrigation treatments were analyzed separately.

Variance components were estimated by restricted maximum likelihood (REML) and heritability as the relationship between genetic and phenotypic variance, according to the formula:

$$h^{2}=\;\frac{\sigma_{g}{}^{2}}{\sigma_{g}{}^{2}+\left(\sigma_{E}{}^{2}/r\right)}$$

BLUPs for genotypes effects are shrinkage predictors that were obtained as:

$$a=GZ^{\prime }V^{-1}\;\left(y-1\mu \right)$$

using matrix notation, where *y* is the vector of the response variable, *G* the matrix of variance covariance of the random effects, *Z′* the design matrix for random effects in the model, *V* estimated variance of *y*, 1 a vector of ones and *μ* the overall mean, the only fixed parameter in the model. For grain yield, anthesis date was included as covariate in the model. Correlations were calculated using BLUPs of the different genotypes using a linear fixed model. Genetic correlations between traits were estimated with a method described previously [38].

### Genotyping and linkage map construction

For all the maize testcrosses phenotyped in this study, leaf samples bulked from 12 plants of each line were used for DNA extraction with a CTAB procedure [39, 40]. DNA of all the samples was sent to the Cornell University Biotechnology Resource Center for GBS (Ithaca, NY, USA). A GBS protocol commonly used by the maize research community was applied in this study [33]. Genomic DNA was digested with the restriction enzyme ApeK1. GBS libraries were constructed in 96-plex, and sequenced on Illumina HiSeq2000. SNP calling and imputation were performed using TASSEL GBS pipeline with B73 as the reference genome [40, 41] to generate a comprehensive genotype collection, the AllZeaGBSv2.7 Production Build (www.panzea.org). This collection includes genotypes of more than 60,000 maize samples. In this study, we focused on the subset of two bi-parental populations, and imputed GBS data was used for further linkage map construction and QTL mapping analyses. Initially, 955,690 SNPs evenly distributed on maize chromosomes were called for each line; 955,120 of them were assigned to chromosomes 1–10, and 570 of them could not be anchored to any of the 10 maize chromosomes. Saturated linkage maps were constructed in QTL IciMapping version 3.2 for each of the bi-parental populations for further QTL mapping analyses, 1,266 and 1,457 SNPs were finally filtered and selected to construct the linkage map in DTPpop and LPSpop, respectively (methods on selecting reliable SNPs and constructing linkage map haven’t been published yet). In the DTPpop, the total length of the linkage map was 1956.92 cM with an average marker density of 1.55 cM. In the LPSpop, the total length of the linkage map was 1813.78 cM with an average marker density of 1.24 cM.

### Multilocation QTL analysis

Inclusive composite interval mapping was used in ICIMv3.2 [42]. 214 and 169 entries were included in mapping for the LPSpop and DTPpop. The walking speed was 1 cM. QTL with a LOD-score above 2.0 were considered to be significant (a = 0.05). QTL for different traits were declared to be collocated when their positions with highest LOD scores (peak) were located in the same bin (IBM2 reference map; maizegdb). Additive (a) and dominance (d) effects for each QTL as estimated with QTL IciMapping v.3.2 were used to calculate the ratio of dominance level (|d/a|). This ratio was used to classify QTL as described previously [43]: additive (A; 0 < = |d/a| < = 0.2); partially dominant (PD; 0.2 < = |d/a| < = 0.8); dominant (D; 0.8 < = |d/a| < = 1.2); overdominant (OD; |d/a| > 1.2). The sign of additive effects was used to identify the origin of the favorable alleles. Positive additivity indicates that the allele increasing trait value was derived from the recurrent parent, while a negative additive effect indicates provenience from the donor. R^2^ represent the total phenotypic variance explained by all QTL detected for individual traits.

## Results

### Summary of phenotypic data and heritability of the target traits

**Fig 1:**
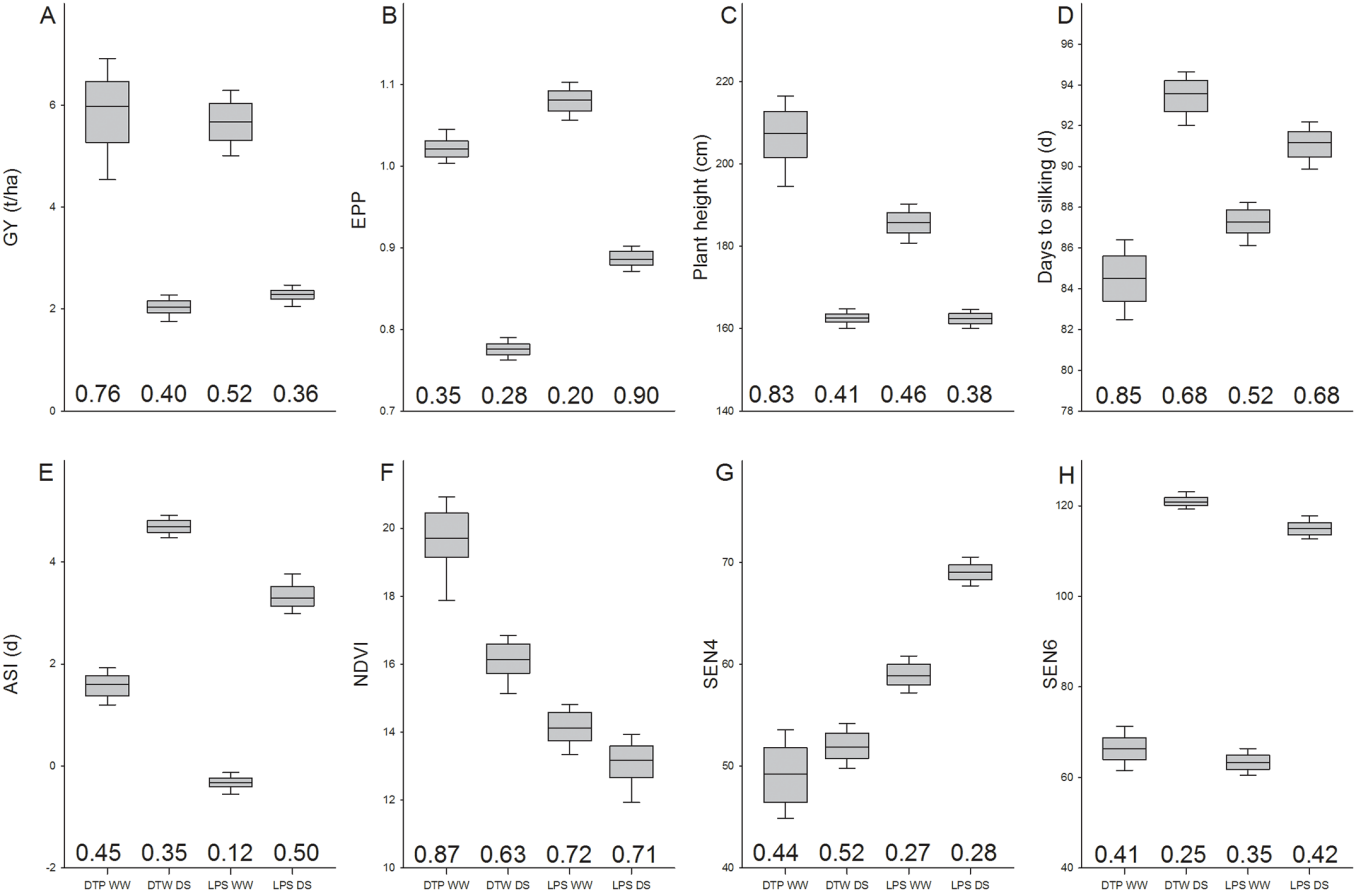
1^st^ quartile, mean, 3^rd^ quartile and heritability for the DTPpop and LPSpop measured under well-watered (WW) and drought stressed conditions (DS). Traits measured were grain yield (GY; A), the number of ears per plant (EPP; B), plant height (PHT; C), silking (D), the anthesis silking interval (ASI; E), NDVI (F) and senescence measured 4 (SEN4; G) and 6 (SEN6; H) weeks after flowering.

The LPSpop and DTPpop were evaluated for grain yield, anthesis, silking, ASI, NDVI, PHT and senescence under well-watered (WW) and drought stressed (DS) conditions. Under well-watered conditions, the interquartile range (IQR) for GY ranged from 5.29 to 6.09 t/ha in the LPSpop (h^2^ = 0.52) and from 5.32 to 6.43 t/ha in the DTPpop (h^2^ = 0.76; Fig 1). Under drought stressed conditions, GY was on average reduced by 65% and 59% in the DTPpop (h^2^ = 0.4) and the LPSpop (h^2^ = 0.36), respectively. Flowering time reflected in the IQR for days to silking ranged from 84.78 to 87.68 in DTPpop (h^2^ = 0.85) and 85.51 to 88.92 in the LPSpop (h^2^ = 0.52) population. Flowering was delayed by 7.92 and 3.81 days under drought stressed conditions compared to well-watered conditions in the DTPpop (h^2^ = 0.68) and the LPSpop (h^2^ = 0.68), respectively. Under well-watered conditions the ASI was generally longer in the DTPpop compared to the LPSpop as indicated by an IQR ranging from 1.38 to 1.83 d for the DTPpop (h^2^ = 0.45) compared to -0.4 to -0.25 d for the LPSpop (h^2^ = 0.11). Under drought stressed conditions the differences between populations were not as significant in the DTPpop (h^2^ = 0.35) relative to the LPSpop (h^2^ = 0.5) since IQR for ASI ranged from 4.58 to 4.82 d in the DTPpop and from 3.13 to 3.41 d for the LPSpop. The DTPpop also showed greater vigor than the LPSpop indicated by higher NDVI values measured before flowering: the IQR for NDVI under well-watered conditions for the DTPpop ranged from 19.61 to 20.84 (h^2^ = 0.87) and from 13.74 to 14.8 for the LPSpop (h^2^ = 0.72). Under drought stressed conditions it ranged from 13.76 to 15.57 for the DTPpop (h^2^ = 0.63) and 12.63 to 13.75 for the LPSpop (h^2^ = 0.71). The DTPpop on average showed greater plant height: The IQR for the DTPpop (h^2^ = 0.83) under well-watered conditions ranged from 201.75 to 212.74 and from 183.36 to 188.18 for the LPSpop (h^2^ = 0.46) while under drought stress it ranged from 161.64 to 163.61 in the DTPpop (h^2^ = 0.42) and from 161.23 to 163.72 in the LPSpop (h^2^ = 0.38). A differential pattern between both populations was measured for visual senescence after flowering. While the DTPpop showed better stay green relative to the LPSpop four weeks after flowering under both conditions the pattern was reversed six weeks after flowering: Under well-watered conditions, IQR for SEN increased from 46.43–50.74 (h^2^ = 0.44) after flowering to 63.97–68.29 six weeks (h^2^ = 0.41) after flowering in the DTPpop. Meanwhile under drought stressed conditions senescence had increased as indicated by IQR ranging from 50.76–53.05 (h^2^ = 0.53) to 120.06–122.07 (h^2^ = 0.25) at four and six weeks after flowering. At the same time the IQR for SEN increased from 55.3–62.975.02–76.5 four weeks after flowering (h^2^ = 0.27) to 57.47–69.2 six weeks (h^2^ = 0.35) after flowering under well-watered conditions and from 65.5–73.49 (h^2^ = 0.28) to 109.5–123.58 (h^2^ = 0.42) under drought stressed conditions in the LPSpop. EPP was generally higher for the LPSpop relative to the DTPpop across irrigation treatments: EPP ranged from 1.01 to 1.03 (h^2^ = 0.35) in the DTPpop and from 1.07–1.09 in the LPSpop (h^2^ = 0.20) under well-watered conditions and from 0.77–0.78 for the DTPpop (h^2^ = 0.26) and 0.87–0.89 in the LPSpop (h^2^ = 0.21) under drought stressed conditions.

### Genetic and phenotypic correlations

A negative (r = -0.31) genetic correlation under drought stressed conditions and a positive correlation (r = 0.32; Table 1) under well-watered conditions were identified in the LPSpop between ASI and grain yield. No significant phenotypic correlations between ASI and grain yield were established determining the need to identify new secondary traits for use in selection for drought tolerance. However, one new secondary trait showing a positive association with grain yield was NDVI. Positive genotypic and phenotypic correlations of NDVI and PHT with grain yield were identified across populations and treatments (r ranging from 0.61 to 0.96). This is indicative of the importance of early vigor (and generally vigor) for plant performance, highlighting the suitability of this trait for selection to increase performance under drought. SEN6, another promising secondary trait, showed a negative genetic and phenotypic (r = -0.49) correlation under drought stressed conditions in the DTPpop while no significant correlation was observed in any other population-by-treatment combination. This illustrates the beneficial effects of improved stay green on grain yield under drought stressed conditions. A positive phenotypic correlation was observed between SEN4 (r = 0.55), SEN6 (r = 0.51) and grain yield under well-watered conditions in the DTPpop indicating the importance of dry-down/ senescence for the re-allocation of nutrients to the growing ear. A positive phenotypic correlation (r = 0.41) was found between EPP and grain yield under drought stressed conditions in the DTPpop, yet surprisingly a negative genetic correlation (r = -0.386) was detected between EPP and grain yield under drought stress in the LPSpop, whereas the phenotypic correlation was positive (r = 0.35). As was to be expected a negative genetic (DTPpop: r = -0.31; LPSpop: r = -0.18) and phenotypic (DTPpop: r = -0.23; LPSpop: r = -0.05) correlation was measured under drought stress in both populations between time to male flowering and grain yield, which is indicative of the importance of drought escape, despite adjusting grain yield for anthesis date.

**Table 1:** Phenotypic (Pheno) and genotypic (Geno) correlations between grain yield and senescence measured 4 (SEN4) and 6 (SEN6) weeks after flowering, the numbers of ears per plant (EPP), NDVI4, the anthesis silking interval (ASI), plant height (PHT) and anthesis.

	DTPpop WW		DTPpop DS		LPSpop WW		LPSpop DS	
	Pheno	Geno	Pheno	Geno	Pheno	Geno	Pheno	Geno
SEN4	0.55		0.21	0.20	0.26		-0.09	0.03
SEN6	0.51		-0.44	-0.859	0.18		-0.18	-0.08
NDVI4	0.71	1.00	0.53	1.00	0.63	1.00	0.73	0.96
EPP	-0.11	0.11	0.26	0.41	0.20	0.38	0.35	-0.38
ASI	0.06	0.11	-0.01	0.02	0.17	0.32	0.01	-0.31
PHT	0.66	0.74	0.44	0.82	0.64	0.87	0.34	-0.21
Anthesis	-0.03	0.06	-0.23	-0.313	0.16	0.57	-0.05	-0.18

### Overdominant QTL

A total of 63 QTL and 42 QTL, of which 26 and 27 overdominant were detected in the LPSpop and DTPpop, respectively (Table 2). 12 overdominant QTL were found for SEN6, ten for EPP, nine for SEN4, seven for ASI, six for NDVI and PHT and three for grain yield. Collocations identified in bins 1.03 and 9.02 indicate the presence of the genetic control for stay green mediated by heterosis. Bin 1.03 harbored overdominant QTL for ASI (WW) and SEN6 (DS) in the LPSpop and for SEN4 (WW) and SEN6 (WW) in the DTPpop while QTL for SEN4 (DS/WW) and SEN6 (DTPpop only/WW) were also found across populations in bin 9.02.

**Table 2:** Summary table of all overdominant QTL measured in the DTPpop (DTP) and LPSpop (LPS) under well-watered (WW) and drought stressed (DS) conditions showing position (Pos), bin, flanking markers, LOD scores, phenotypic variance explained by a QTL (PVE), additive and dominance effects. Traits measured are senescence 4 (SEN4) and 6 (SEN6) weeks after flowering, NDVI, grain yield (GY), ears per plants (EPP), anthesis, the anthesis silking interval (ASI) and plant height (PHT).

Pop	Trait	Chr	Pos (cM)	Pos (cM)	Bin	LeftMarker	RightMarker	LOD	PVE(%)	Add	Dom	D/A
LPS	SEN6_WW	1	105.2	105.2	1.03	S1_46929837	S1_45930625	2.69	4.74	0.07	-1.01	-14.68
LPS	SEN4_WW	1	105.2	105.2	1.03	S1_46929837	S1_45930625	2.60	4.26	0.05	-0.62	-12.91
DTP	ASI_WW	1	178.7	181.0	1.03	S1_45005937	S1_44329708	2.48	6.98	0.56	2.23	3.96
DTP	SEN6_DS	1	196.5	197.0	1.03	S1_33953448	S1_34826690	2.28	5.96	0.43	2.21	5.17
LPS	ASI_DS	1	127.9	129.7	1.04	S1_69287246	S1_68134904	3.03	5.17	0.00	0.14	-39.58
DTP	SEN6_WW	1	138.1	140.4	1.04	S1_81105620	S1_77150339	2.20	6.13	-0.12	0.70	-5.76
DTP	SEN6_DS	1	95.9	95.9	1.05	S1_171740910	S1_175645169	3.08	9.32	-0.15	-0.48	3.30
LPS	NDVI4_WW	1	286.2	286.2	1.07	S1_213232490	S1_214308454	2.33	4.07	-0.12	-0.29	2.34
LPS	SEN4_DS	1	386.1	387.9	1.11	S1_287291295	S1_288015387	2.62	5.19	0.06	-0.56	-8.71
LPS	SEN6_DS	1	386.1	387.9	1.11	S1_287291295	S1_288015387	2.77	5.87	-0.05	-1.23	22.60
DTP	PHT_WW	2	302.5	302.9	2.01	S2_2680540	S2_1353401	2.24	5.93	-0.06	-1.19	18.42
DTP	ASI_DS	2	1.6	3.0	2.04	S2_44435431	S2_45987592	2.01	5.18	-0.02	-0.10	4.19
DTP	GY_WW	2	183.2	186.3	2.05	S2_105547777	S2_140751716	2.72	6.80	0.39	-0.75	-1.94
DTP	NDVI4_DS	2	175.9	177.5	2.07	S2_176725918	S2_175753923	2.63	7.30	-0.16	-0.44	2.77
DTP	SEN4_DS	2	129.1	131.8	2.1	S2_236486108	S2_234801048	2.31	6.46	-0.26	0.57	-2.17
LPS	ASI_DS	3	61.2	65.1	3.07	S3_177679284	S3_205474551	2.79	5.97	-0.01	0.08	-7.64
LPS	EPP_DS	3	94.2	95.5	3.09	S3_223175381	S3_223539069	2.05	3.40	0.00	-0.01	51.00
LPS	EPP_DS	3	107.9	107.9	3.09	S3_225898308	S3_226975720	2.34	4.44	0.00	-0.01	-31.00
LPS	GY_DS	3	116.1	126.1	3.09	S3_228264712	S3_229247257	2.01	3.91	0.01	-0.04	-3.02
DTP	SEN6_WW	4	39.8	42.1	4.01	S4_138427	S4_2514397	2.62	7.47	0.23	-2.05	-8.81
DTP	SEN6_DS	4	42.1	43.4	4.01	S4_2514397	S4_3261039	2.29	6.40	-0.12	-0.92	7.70
DTP	SEN6_DS	4	49.1	66.4	4.01	S4_3945335	S4_4622784	2.20	6.12	-0.07	-0.89	13.33
LPS	SEN4_DS	4	171.2	171.9	4.05	S4_146977468	S4_154047619	2.74	5.47	-0.05	-0.69	13.07
LPS	NDVI4_DS	4	121.2	121.6	4.06	S4_170468004	S4_169557853	2.06	3.30	0.15	0.37	2.46
DTP	EPP_DS	4	132.0	132.9	4.08	S4_155912460	S4_161045008	3.26	8.91	0.00	0.01	-3.59
DTP	EPP_WW	4	161.3	162.1	4.08	S4_187604350	S4_188521835	2.33	6.51	0.00	0.01	-2.90
LPS	ASI_DS	5	1.3	6.7	5	S5_536180	S5_1232105	2.25	3.47	0.01	-0.11	-7.79
LPS	PHT_DS	5	10.3	17.2	5	S5_2521499	S5_3258461	2.14	2.89	0.59	-0.71	-1.20
LPS	ASI_DS	5	17.2	17.2	5.01	S5_3258461	S5_4668442	2.05	3.54	-0.03	-0.13	4.43
LPS	SEN6_DS	5	194.1	194.1	5.03	S5_69125858	S5_76217531	2.19	4.42	0.33	1.22	3.69
DTP	EPP_DS	5	127.0	127.0	5.04	S5_205840142	S5_207168426	2.27	5.82	0.00	-0.01	2.86
LPS	PHT_DS	5	80.6	80.6	5.06	S5_202801986	S5_203803707	3.22	4.48	0.10	-1.69	-17.46
LPS	EPP_DS	6	146.1	146.5	6.01	S6_22918484	S6_25741733	2.17	3.30	0.00	0.01	4.04
LPS	EPP_DS	6	127.8	129.8	6.02	S6_127200702	S6_61815312	2.21	17.64	0.01	0.02	3.00
DTP	EPP_DS	6	46.4	47.9	6.04	S6_153880358	S6_153267024	2.18	6.26	0.00	0.01	-29.00
DTP	PHT_DS	6	46.4	47.9	6.04	S6_153880358	S6_153267024	3.65	10.87	-0.17	1.26	-7.49
DTP	SEN6_WW	6	91.7	91.7	6.04	S6_118704242	S6_112005588	2.54	6.88	-0.79	-2.28	2.91
DTP	SEN4_WW	6	91.7	91.7	6.04	S6_118704242	S6_112005588	2.82	7.64	-0.64	-2.18	3.38
LPS	PHT_DS	6	45.4	45.4	6.06	S6_158261606	S6_155067595	2.93	4.64	0.44	1.02	2.34
DTP	GY_WW	7	169.5	172.4	7.01	S7_9332272	S7_8050670	2.03	5.96	-0.27	-0.57	2.09
DTP	NDVI4_WW	7	0.4	0.4	7.05	S7_175034148	S7_174586481	2.18	5.64	0.14	-0.31	-2.20
LPS	SEN4_DS	8	116.1	117.3	8	S8_2839072	S8_2080697	2.10	4.37	-0.33	-0.67	2.04
DTP	SEN6_DS	8	205.3	205.3	8.01	S8_3629473	S8_873690	2.09	6.13	-0.11	-0.49	4.36
DTP	NDVI4_DS	8	205.3	205.3	8.01	S8_3629473	S8_873690	2.30	5.03	-2.04	-3.24	1.59
DTP	PHT_WW	8	29.8	31.3	8.06	S8_159989296	S8_160612253	2.25	6.28	0.03	0.05	1.76
DTP	SEN4_WW	9	154.2	154.2	9.01	S9_8039883	S9_7268409	2.48	7.05	0.06	-0.44	-7.14
LPS	SEN6_WW	9	32.8	35.9	9.02	S9_18335081	S9_17724665	2.94	5.27	0.09	1.35	15.02
LPS	SEN4_WW	9	35.9	43.2	9.02	S9_17724665	S9_16326906	3.88	6.60	0.10	0.98	9.96
DTP	SEN4_DS	9	138.8	138.8	9.02	S9_17969570	S9_16739269	2.59	7.65	0.07	1.13	16.41
LPS	EPP_DS	9	50.0	51.3	9.03	S9_45707438	S9_76791785	4.17	6.20	-0.01	-0.01	1.68
LPS	EPP_DS	9	58.3	58.6	9.03	S9_56424613	S9_48441455	4.70	6.95	-0.01	-0.01	1.97
DTP	ASI_DS	10	60.4	60.4	10.03	S10_114353956	S10_2839576	2.34	6.03	0.00	0.01	2.35
LPS	NDVI4_WW	10	111.2	111.8	10.04	S10_144739622	S10_145352088	2.47	4.31	-0.15	-0.28	1.90

A vigor QTL (measured as PHT under DS) with potential effects on stay green (WW, SEN4, SEN6) and EPP (DS) was found in bin 6.04. Vigor QTL (measured as NDVI) with potential effects on stay green (SEN6) were detected in bins 8.01 and 2.07 in the DTPpop. Another collocation for stay green (SEN4, SEN6) was found in the DTPpop in bin 1.11.

### Detected non-overdominant QTL

In the LPSpop six QTL were found for EPP, grain yield, and NDVI, four for anthesis and senescence, nine for PHT and two for ASI with LOD scores ranging from 2.02 (SEN4, bin 1.07) to 17.2 (anthesis, bin 2.06) at a PVE of 3.78 and 26.02%, respectively. In the DTPpop four QTL were identified for anthesis and grain yield, three for SEN, two for PHT and one for NDVI and EPP each with LOD scores ranging from 2.17 (PHT, bin 2.09) to 12.9 (anthesis, bin 2.06) at PVE of 17.1% and 31.28%, respectively (Table 3).

**Table 3:** Summary table of all non-overdominant QTL measured in the DTPpop (DTP) and LPSpop (LPS) population under well-watered (WW) and drought stressed (DS) conditions showing position (Pos), bin, flanking markers, LOD scores, phenotypic variance explained by a QTL (PVE), additive and dominance effects. Traits measured are senescence measured 4 (SEN4) and 6 (SEN6) weeks after flowering, NDVI, grain yield (GY), ears per plants (EPP), anthesis, the anthesis silking interval (ASI) and plant height (PHT).

Population	Trait	Chr	Pos (cM)	Pos (cM)	Bin	LeftMarker	RightMarker	LOD	PVE(%)	Add	Dom	D/A
LPS	GY_WW	1	17.8	20.9	1.01	S1_5089063	S1_5877060	4.14	7.36	0.20	0.05	0.27
LPS	NDVI_DS	1	45.7	48.5	1.02	S1_16469352	S1_17963080	2.82	4.66	0.28	0.09	0.34
LPS	PHT_WW	1	47.9	49.9	1.02	S1_19133052	S1_19791935	13.35	21.08	2.46	-0.08	-0.03
DTP	SEN6_WW	1	225.8	228.9	1.02	S1_21722506	S1_19348057	2.23	7.36	-0.73	-0.42	0.58
LPS	NDVI_WW	1	300.0	306.2	1.02	S1_222400848	S1_224102167	5.04	9.34	0.19	-0.21	-1.08
LPS	PHT_DS	1	102.0	105.0	1.03	S1_43770018	S1_45405135	5.04	8.54	0.69	-0.13	-0.19
DTP	SEN6_WW	1	128.8	135.8	1.05	S1_88531733	S1_80185124	2.65	7.39	0.10	-0.02	-0.19
LPS	ASI_DS	1	228.1	231.1	1.05	S1_176845338	S1_179724425	10.91	19.47	0.19	0.00	0.00
LPS	SEN4_DS	1	261.2	263.9	1.07	S1_204866026	S1_203170951	2.02	3.78	0.34	0.20	0.59
LPS	PHT_WW	1	382.9	387.1	1.1	S1_284548803	S1_287291295	3.88	5.42	-1.19	-0.15	0.12
DTP	NDVI_DS	1	7.8	12.3	1.11	S1_293954732	S1_299122254	2.27	6.34	1.55	1.30	0.84
LPS	AD_DS	1	377.57	378.88	1.11	S1_285986869	S1_285536326	6.933	9.2329	0.372	0.065	1.332
LPS	NDVI_DS	1	397.7	401.1	1.11	S1_292336703	S1_293223070	3.75	6.34	-0.23	0.10	-0.41
LPS	NDVI_DS	2	192.4	195.7	2.02	S2_17790055	S2_13864396	4.79	7.89	0.27	-0.02	-0.09
LPS	SEN4_WW	2	192.4	195.7	2.02	S2_17790055	S2_13864396	3.99	7.58	0.51	-0.09	-0.17
LPS	SEN6_WW	2	192.4	195.7	2.02	S2_17790055	S2_13864396	3.89	7.13	0.78	-0.06	-0.07
LPS	EPP_WW	2	192.4	195.7	2.02	S2_17790055	S2_13864396	4.02	8.64	-0.01	0.00	-0.41
LPS	GY_WW	2	183.9	185.9	2.03	S2_21792369	S2_18766037	2.34	3.69	0.12	0.06	0.47
LPS	PHT_WW	2	153.7	156.1	2.04	S2_50022058	S2_54093869	10.81	16.35	2.09	0.35	0.17
LPS	ASI_DS	2	169.7	172.3	2.04	S2_41024393	S2_40148342	2.89	4.77	0.08	-0.01	-0.14
LPS	EPP_DS	2	170.3	172.9	2.04	S2_40148342	S2_38765165	6.18	11.15	-0.01	0.00	0.10
LPS	PHT_DS	2	107.5	110.1	2.05	S2_149517847	S2_100637635	4.72	8.11	0.65	-0.19	-0.29
LPS	AD_WW	2	116.76	118.13	2.05	S2_96491914	S2_91854914	7.886	14.8716	0.311	0.084	1.886
LPS	EPP_WW	2	148.7	152.4	2.05	S2_54765244	S2_60276380	12.62	20.27	-0.01	0.00	-0.10
LPS	AD_DS	2	125.62	125.95	2.06	S2_169736203	S2_172863406	17.09	26.0015	0.577	-0.12	1.521
DTP	AD_WW	2	168.03	169.04	2.06	S2_185378123	S2_180527543	12.85	31.2834	0.135	-1.59	2.435
DTP	SEN6_DS	2	64.3	66.3	2.07	S2_188900919	S2_192855248	9.14	23.65	4.99	-1.99	-0.40
DTP	PHT_WW	2	65.1	68.0	2.07	S2_193882787	S2_194494239	2.30	5.99	0.27	-0.08	-0.30
DTP	GY_WW	2	157.4	159.4	2.07	S2_191003187	S2_191973122	2.30	6.19	0.30	0.08	0.26
DTP	PHT_DS	2	155.2	157.2	2.09	S2_232596221	S2_190540940	2.17	17.14	1.38	1.43	1.04
LPS	GY_DS	2	11.5	13.5	2.1	S2_229478190	S2_228175085	2.66	5.23	-0.04	-0.03	0.66
LPS	GY_DS	4	217.5	219.8	4.03	S4_21137086	S4_20100785	3.99	8.31	-0.05	-0.01	0.29
LPS	PHT_DS	4	118.3	121.0	4.06	S4_171266260	S4_167184961	2.03	3.21	0.59	0.52	0.87
LPS	EPP_DS	5	9.3	18.2	5	S5_2521499	S5_3258461	2.34	4.18	0.00	0.00	0.29
LPS	NDVI_DS	5	197.9	202.8	5.02	S5_82956371	S5_64808654	11.25	19.92	0.49	-0.01	-0.01
LPS	EPP_DS	5	244.7	246.7	5.02	S5_11066420	S5_10279634	3.46	6.12	0.01	0.00	0.04
LPS	EPP_DS	5	146.0	148.0	5.04	S5_163295270	S5_159804181	4.98	8.91	0.00	0.00	-0.38
LPS	NDVI_WW	5	156.5	159.2	5.04	S5_133334845	S5_136416259	2.73	5.57	0.20	-0.06	-0.28
LPS	GY_WW	5	156.5	159.2	5.04	S5_133334845	S5_136416259	10.27	18.63	0.29	0.03	0.09
LPS	PHT_WW	5	185.5	188.5	5.04	S5_140262152	S5_142723254	2.22	2.88	0.97	0.75	0.77
LPS	SEN4_WW	6	154.2	156.2	6	S6_2058763	S6_780111	2.65	4.46	0.46	0.08	0.18
DTP	AD_DS	7	180.48	184.31	7.01	S7_5612646	S7_5106703	2.437	5.6878	0.479	0.468	2.334
DTP	GY_DS	8	26.2	28.6	8.06	S8_162563112	S8_161544717	2.80	7.81	-0.58	-0.62	1.07
DTP	GY_WW	8	26.2	28.6	8.06	S8_162563112	S8_161544717	2.61	7.49	0.27	0.25	0.94
DTP	GY_WW	9	105.2	107.6	9.03	S9_94790194	S9_82283843	2.14	5.60	1.10	1.00	0.91
DTP	AD_DS	9	95.55	97.7	9.04	S9_106201455	S9_33138292	3.643	8.5928	0.495	0.277	2.359
DTP	AD_WW	9	128.37	128.77	9.05	S9_24926377	S9_25258536	4.795	9.9837	0.632	0.522	2.082
LPS	AD_WW	9	147.13	147.8	9.07	S9_149010372	S9_151368606	3.087	5.4526	0.203	0.068	1.766
DTP	EPP_DS	10	22.1	24.9	10.04	S10_141649268	S10_141128782	2.69	7.45	0.07	0.00	-0.02
LPS	PHT_DS	10	70.7	73.0	10.04	S10_108059601	S10_113021442	5.90	9.92	-0.66	0.55	-0.83
LPS	PHT_WW	10	76.2	78.5	10.04	S10_127258661	S10_129348068	5.15	7.33	-1.53	-0.20	0.13
LPS	GY_WW	10	77.8	81.2	10.04	S10_128698701	S10_127622737	3.15	5.27	-0.17	-0.07	0.41

### Phenotypic variation explained by all QTL for a trait (R^2^)

Under well-watered conditions variation (R^2^) explained by all QTL (excluding overdominant QTL) for a single trait ranged from 5.2 (ASI) to 36.6% (PHT) in the LPSpop and from 0 (NDVI4) to 28.2% (Anthesis) in the DTPpop (Table 4). Under drought stressed conditions R^2^ ranged from 5.2 (SEN6) to 42.5% (Anthesis) in the LPSpop and from 0 (NDVI) to 23.8% (Anthesis) in the DTPpop. Low R^2^ can be explained by low heritability for certain traits (e.g. ASI) or the genetic control of a trait by multiple QTL with small effects, that could not be detected in this study, when low R^2^ coincided with high heritability (e.g. grain yield, NDVI).

**Table 4:** R^2^ values measured in the DTPpop and LPSpop under well-watered and drought stressed conditions. Traits displayed are: grain yield (GY), anthesis, senescence measured 4 (SEN4) and 6 (SEN6) weeks after flowering, NDVI4 measured six weeks after emergence, the anthesis silking interval (ASI), plant height (PHT) and the numbers of ears per plant (EPP).

Trait	DTPpop-WW	DTPpop-RR	LPSpop-WW	LPSpop-RR
GY	6.24	0.00	24.83	7.52
Anthesis	28.16	23.38	18.6	42.45
SEN4	6.54	6.84	24.36	10.61
SEN6	6.32	0.00	19.41	5.29
NDVI4	0.00	12.63	16.53	26.54
ASI	6.79	6.85	5.22	25.63
PHT	21.92	8.76	36.63	24.43
EPP	0.00	7.24	25.89	21.38

### Identification of early vigor QTL with potential effects on grain yield and yield components

Across both populations a total of 18 QTL for either NDVI or PHT indicative of plant vigor were detected in both populations (Table 3). Out of the 18 vigor QTL, the trait increasing allele was derived from CML491 14 times, indicative of the importance of the alleles derived from CML491 for a rapid crop establishment and fast vegetative growth. A total of eight QTL for flowering time were detected in both populations across treatments in bins 1.11, 2.05, 2.06, 7.01, 9.04, 9.05 and 9.07. In all cases the allele derived from CML491 delayed flowering. Considering high PVE and negative correlation of flowering time with grain yield these QTL seem promising to develop germplasm flowering earlier potentially escaping drought.

**Fig 2:**
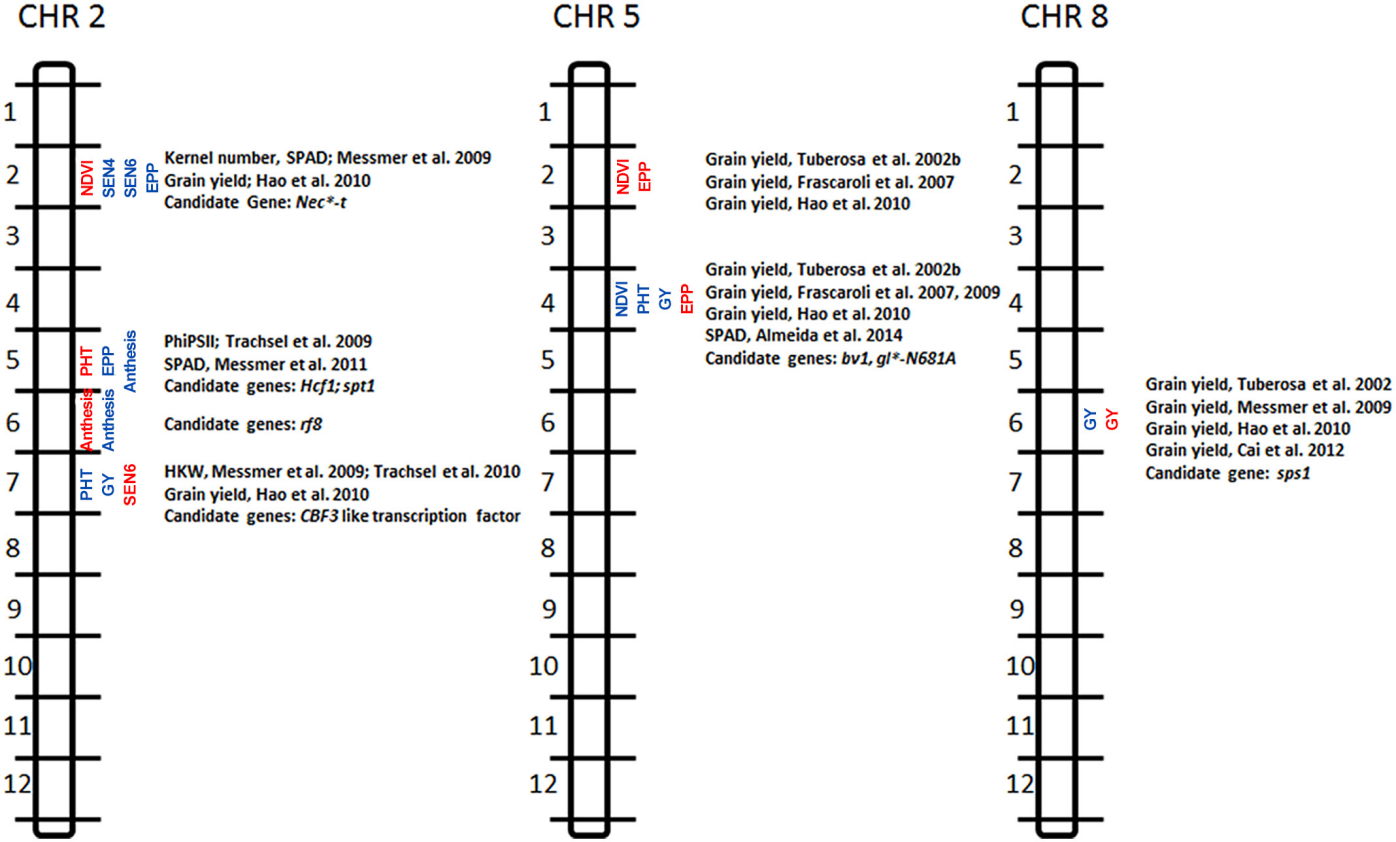
Most important QTL for vigor, flowering and grain yield identified in well-watered (blue font) and drought stressed conditions (red font) in bins 2.02, 2.05, 2.06, 2.07, 5.02, 5.04 and 8.06 in the LPSpop and DTPpop, collocations with QTL for related traits in other studies and underlying candidate genes.

Seven QTL cluster identified for early vigor (bins 2.02, 2.05, 2.07, 5.02, 5.04), flowering time (bin 2.06) and grain yield (8.06) deserve further attention for validation and potential use in marker assisted selection based on high PVE, identification across populations and/ or environmental conditions and their strong association with grain yield (Fig 2).

### QTL for early vigor

The collocation of QTL for NDVI (WW), PHT (WW), GY (WW) and EPP (DS) in the LPSpop in bin 5.04 suggests that this locus has concomitant effects on vigor and grain yield under well-watered conditions and EPP under drought stressed conditions. While the allele increasing vigor (NDVI, WW; +0.2) and grain yield (0.29 t/ha) under well-watered conditions was derived from CML491, the allele increasing EPP by 0.0018 under drought stressed conditions was derived from LPSC7F64. It is worth noting that the QTL for NDVI explained 5.5%, while the QTL for grain yield explained 18.6% of the observed phenotypic variation.

Potential effects of a chromosomal region on vigor, stay-green and EPP were detected in bin 2.02 where a QTL for NDVI detected under drought stressed conditions collocated with a QTL for EPP and two QTL for senescence under well-watered conditions. The allele derived from CML491 increased vigor (NDVI, DS: +0.27), while the allele derived from LPSC7F64 increased EPP by 0.01. The allele derived from LPSC7F64 equally reduced senescence by 0.51 and 0.78. LOD scores for the QTL for EPP reached 4.02 explaining 8.64% of the observed phenotypic variance.

Bin 5.02 harbored two QTL for NDVI and EPP under drought stress in the LPSpop. The allele derived from CML491 increased NDVI and EPP by 0.49 and 0.01, respectively indicative of a common genetic control. LOD scores and PVE for the QTL for NDVI reached 11.25 and 19.9%, respectively.

The vigor QTL (PHT DS) detected under drought in bin 2.05 collocated with two QTL for EPP and anthesis under well-watered conditions. The allele derived from CML491 increased PHT by 0.65 cm and delayed flowering by 0.31 d, while the allele derived from LPSC7F64 increased EPP by 0.01; QTL for EPP and time to flowering had LOD scores of 12.6 and 7.89 explaining 20.27% and 14.87% of the observed phenotypic variance.

A vigor QTL (PHT WW) in bin 2.07 measured under well-watered conditions collocated with a QTL for senescence under drought stress and a QTL for grain yield under well-watered conditions in the DTPpop. Similar to PHT (+0.27 cm), grain yield was increased by the allele derived from CML491 by 0.3 t/ha, while the allele derived from DTPWC9F104 reduced senescence by 4.99 explaining 23.65% of the observed phenotypic variance. It should be noted that an overdominant QTL for NDVI was detected in the same bin under well-watered conditions, further emphasizing the importance of this locus for the genetic control of vigor.

### Constitutive grain yield QTL in bin 8.06

In the DTPpop, bin 8.06 harbored a constitutive QTL for grain yield (independent of early vigor) under well-watered and drought stressed conditions with potential interest for marker assisted selection. The allele derived from DTPWC9F104 increased grain yield by 0.58 t/ha under drought stressed conditions while the allele derived from CML491 increased grain yield by 0.27 t/ha under well-watered conditions. Both QTL explained 7.8 and 7.5% of the observed phenotypic variance.

### Flowering time QTL in bin 2.06

Two strong anthesis QTL mapped to bin 2.06 in the DTPpop and the LPSpop under well-watered and drought stressed conditions. QTL had LOD scores of 12.9 and 17.1 explaining 31.2% and 26% of the phenotypic variation observed. Similar to the other six anthesis QTL, the allele derived from CML491 delayed flowering by 0.14 d and 0.58 d in the DTPpop and the LPSpop, respectively.

### Hybrids with best performance under WW and DS

Grain yield in the DTPpop and LPSpop was significantly affected by factors, genotype, irrigation treatment and the genotype-by-environment interaction (data not shown). As a result the ranking of genotypes changed across irrigation treatments (Table 5). In all treatment-by-population combination (except LPSpop WW) the best ten entries evaluated in these trials out-yielded the best check (CML312/CML444) by 32.5% (DTPpop WW) to 60% (DTPpop DS) indicating the suitability of testcross/lines evaluated here to increase drought tolerance in breeding germplasm. As a result of the strong genotype-by-environment interaction only one (((CML491/DTPWC9F104)//CML491)B2/CML503) and two entries (((CML491/LPSC7F64)//CML491)B154/CML503; ((CML491/LPSC7F64)//CML491)B218/CML503) ranked in the top 10 under well-watered and drought stressed conditions in the DTPpop and LPSpop, respectively. These lines represent significantly improved and partially representative CML491 versions that could be used to either develop large number of experimental hybrid combinations or as donors for drought tolerance in the low-land tropics breeding program without yield penalty under optimal conditions.

**Table 5:** Grain yield (GY) for the best lines and best check (CML312/CML444) measured in the LPSpop (LPS) and DTPpop (DTP) under well-watered and drought stressed conditions.

	**Well watered**		**Drought stressed**	
		**GY**		**GY**
DTP	**Hybrid**	**t/ha**	**Hybrid**	**t/ha**
	((CML491/DTPWC9F104)//CML491)B3/CML503	8.48	((CML491/DTPWC9F104)//CML491)B76/CML503	2.94
	((CML491/DTPWC9F104)//CML491)B10/CML503	7.96	((CML491/DTPWC9F104)//CML491)B86/CML503	2.74
	((CML491/DTPWC9F104)//CML491)B6/CML503	7.93	((CML491/DTPWC9F104)//CML491)B77/CML503	2.65
	((CML491/DTPWC9F104)//CML491)B104/CML503	7.92	((CML491/DTPWC9F104)//CML491)B47/CML503	2.47
	((CML491/DTPWC9F104)//CML491)B96/CML503	7.76	((CML491/DTPWC9F104)//CML491)B108/CML503	2.47
	((CML491/DTPWC9F104)//CML491)B128/CML503	7.75	((CML491/DTPWC9F104)//CML491)B2/CML503	2.42
	((CML491/DTPWC9F104)//CML491)B120/CML503	7.74	((CML491/DTPWC9F104)//CML491)B63/CML503	2.40
	((CML491/DTPWC9F104)//CML491)B172/CML503	7.59	((CML491/DTPWC9F104)//CML491)B53/CML503	2.39
	((CML491/DTPWC9F104)//CML491)B2/CML503	7.58	((CML491/DTPWC9F104)//CML491)B24/CML503	2.37
	((CML491/DTPWC9F104)//CML491)B99/CML503	7.52	((CML491/DTPWC9F104)//CML491)B32/CML503	2.36
	**Best Check**	6.40	**Best Check**	1.81
		**GY**		**GY**
LPS	**Hybrid**	**t/ha**	**Hybrid**	**t/ha**
	CML312/CML444	8.29	((CML491/LPSC7F64)//CML491)B85/CML503	3.39
	((CML491/LPSC7F64)//CML491)B154/CML503	8.15	((CML491/LPSC7F64)//CML491)B236/CML503	3.38
	((CML491/LPSC7F64)//CML491)B108/CML503	7.75	((CML491/LPSC7F64)//CML491)B154/CML503	3.35
	((CML491/LPSC7F64)//CML491)B104/CML503	7.35	((CML491/LPSC7F64)//CML491)B102/CML503	3.04
	((CML491/LPSC7F64)//CML491)B118/CML503	7.33	((CML491/LPSC7F64)//CML491)B218/CML503	2.95
	((CML491/LPSC7F64)//CML491)B218/CML503	7.30	((CML491/LPSC7F64)//CML491)B119/CML503	2.95
	((CML491/LPSC7F64)//CML491)B91/CML503	7.29	((CML491/LPSC7F64)//CML491)B79/CML503	2.92
	((CML491/LPSC7F64)//CML491)B146/CML503	7.22	((CML491/LPSC7F64)//CML491)B136/CML503	2.92
	((CML491/LPSC7F64)//CML491)B200/CML503	7.19	((CML491/LPSC7F64)//CML491)B143/CML503	2.90
	((CML491/LPSC7F64)//CML491)B149/CML503	7.19	((CML491/LPSC7F64)//CML491)B82/CML503	2.87
	**Best Check**	8.29	**Best Check**	2.46

## Discussion

In the current study we aimed to identify QTL for secondary traits associated with grain yield in two BC_1_F_2:3_backcross populations under well-watered and drought stressed conditions. Averaged across locations mean grain yield reached 5.6 t/ha and 5.8 t/ha under well-watered conditions in the LPSpop and the DTPpop, respectively. Drought stress reduced grain yield by 60–65% relative to the non-stressed control, indicating that measured grain yield can be attributed to drought tolerance as suggested previously [44], but that ‘yield carry over’ as a result of residual vigor cannot be ruled out. It is not clear why a greater number of QTL was found in the LPSpop (63) population compared to the DTPpop (42). We speculate that differences in population size (LPSpop: 220 entries; DTPpop: 175) may have contributed to this finding. Based on phenotypic and genotypic data acquired in this study we can rule out differences in the quality of phenotypic and genotypic data, or marker density to be accountable. We detected a total of 53 overdominant QTL (26 in the LPS and 27 in the DTPpop) as was to be expected using an BC_1_F_2:3_ backcross population structure. It is not clear to what extent overdominant QTL detected may be masking additive/partially dominant QTL with less strong effects, as the inbred line populations were not evaluated phenotypically in the current study.

In contrast to most other studies carried out under drought stressed conditions [12, 14, 28, 45, 46] only weak correlations were observed between ASI and grain yield. Similarly, only two weak QTL for ASI (both in the LPSpop) were detected. Seeming discrepancies to earlier studies can be explained by the population structure used in the current study and longterm selection against long ASI [47] in CIMMYT breeding programs. In contrast to older studies where populations with a tolerant and a susceptible parent were used [14], the recurrent parent in both populations evaluated in this study was an advanced elite line that had undergone selection for tolerance to drought. Smaller phenotypic variation for ASI had therefore been expected. The lack/ reduction of variance for traits used in selection in the past re-emphasizes the need to identify new (secondary) traits allowing to improve performance under drought stress.

A secondary trait suitable for selection should be fast, easy and cheap to measure, have a high heritability (if possible higher than grain yield) and a strong association with grain yield [17]. One such trait showing a strong correlation with grain yield under well-watered (LPSpop: r = 0.63; DTPpop: r = 0.71) and drought stressed conditions (LPSpop: r = 0.73; DTPpop: r = 0.71) with heritability higher than grain yield in both populations is NDVI. Early vigor as indicated by NDVI is indicative of a plant’s ability to suppress weeds during crop establishment and to form a deep(er) root system [48] allowing them to efficiently uptake water and nitrogen [47, 48] from deep soil strata over the course of the cropping season [49, 50, 51]. If water and nutrients additionally acquired by means of a deeper root system and nutrients are not offset by larger shoots, resulting increased transpiration and/or lodging, plants with improved early vigor under drought will yield higher. In this study plants with greater NDVI did indeed yield higher under drought stressed (and well-watered) conditions. Selection for vigor QTL are therefore desirable as long as plant height is not excessively increased and standability is not impaired. Negative correlations detected between time to anthesis and GY in this study (DTPpop: r = -0.18; LPSpop: r = -0.25) indicate that grain yield could be increased by means of an earlier flowering allowing plants to escape drought. Reductions in yield potential expected under optimum conditions, as a result of earlier flowering could be overcome by increasing early vigor as shown previously [19].

Markers/QTL to be considered in marker assisted selection should have high LOD, PVE and R^2^, while traits in question should at the same time be associated with grain yield. Based on these considerations seven chromosomal segments located in bins 2.02, 2.05, 2.06, 2.07, 5.02, 5.04 and 8.06 should be further investigated for their potential use in breeding (Fig 2).

### Early vigor QTL conferring grain yield under well-watered conditions in bin 5.04

The importance of the vigor QTL in bin 5.04 for the control of PHT, NDVI and assimilate partitioning is further emphasized by the collocation with QTL for grain yield in studies carried out [52, 53, 54] in several genetically distinct populations. Collocations with QTL for visual senescence, SPAD [28] and grain yield [26] across different genetic backgrounds furthermore indicate potential effects of this chromosomal segment on the plants ability to grow and yield under drought stressed conditions. Potential candidate genes underlying the observed effects and collocations may be d*-dwarf candidate6, *bv1* (*brevis plant1*) and gl*-N681A (*glossyN681A*). The d*-dwarf candidate6 gene determines plant development mediated by gibberellic acid, *bv1* determines internode length while gl*-N681A determines leaf width. It is therefore conceivable that individual effects or interactions among these genes may explain QTL detected for NDVI and plant height.

### Early vigor QTL conferring high grain yield under drought stressed condition in bin 5.02

The QTL identified under drought for vigor and EPP in bin 5.02 collocated with QTL for grain yield identified by several authors under well-watered [53, 26] and under drought stressed conditions [54] indicative of the potential effect of this chromosomal segment on vigor, assimilate partitioning and resulting grain yield across populations. Considering high LOD scores (11.25), the high phenotypic variation explained (19.92%), and the strong phenotypic/genotypic correlation of NDVI with grain yield, this QTL should be considered for marker assisted selection to increase NDVI, EPP and resulting grain yield under drought stressed conditions. No putative candidate genes could be identified in this chromosomal region. In future research the QTL at this position could be validated by positional cloning.

### Early vigor QTL in bin 2.02 with potential beneficial effects on grain yield

The importance of QTL for early vigor under drought stressed conditions and EPP and stay-green (2 x SEN) under well-watered conditions detected in bin 2.02 is highlighted by the collocation observed with a QTL for kernel number, SPAD [14] and grain yield [26] under drought stressed conditions identified previously in multiple populations. The collocations observed in this bin indicate the presence of a genetic area controlling vigor (under DS) potentially affecting assimilate partitioning to the ear and resulting grain yield. The physiological/genetic interrelation with a QTL detected for EPP, senescence (WW only) under well-watered conditions is not clear. A potential candidate gene underlying the detected QTL may be Nec*-t. Nec*-t is responsible for stress induced reduction of chloroplasts and chlorophyll [55] potentially explaining the observed effects of this chromosomal segment on vigor, senescence, EPP and grain yield.

### Vigor QTL conferring grain yield under drought stressed conditions in bin 2.05

The QTL for vigor detected under drought and for EPP under well-watered conditions in bin 2.05 collocated with QTL for quantum efficiency of photosystem II [29] and leaf chlorophyll content [15] under well-watered conditions in the CML444 x SC-Malawi population highlighting the importance of this segment. Two candidate genes underlying the observed collocations have been identified: *Hcf1* and *spt1*. *Hcf* has been shown to affect the NADP+ oxireductase resulting in efficient electron transport in photosynthesis and radiation use efficiency [56]. *Spt1* has been shown to affect leaf chlorophyll content and general vigor. It is therefore likely that individual or combined action of *hcf1* and/or *spt1* affect leaf chlorophyll content, photosynthesis and resulting vigor under drought stressed conditions as well as assimilate partitioning to the growing ear under well-watered and drought stressed conditions as indicated by the observed collocations.

### Vigor QTL in bin 2.07 conveying grain yield under well-watered conditions

The importance of the vigor QTL detected under well-watered conditions in bin 2.07 is highlighted by the collocations with QTL for the hundred kernel weight and grain yield under well-watered [14, 29] and drought stressed conditions [26] in other populations. The observed collocations indicate a vigor mediated QTL for improved stay green with potential beneficial effects on yield components and grain yield under well-watered and drought stressed conditions. A potential candidate gene underlying the observed effects is a *CBF3* like transcription factor [26]. In arabidopsis *CBF3* like transcription factors have been shown to increase growth and development under low temperatures and drought and modulate the sugar metabolism in the plant [57].

### Grain yield QTL in bin 8.06 under well-watered and drought stressed conditions

The grain yield QTL detected under well-watered and drought stressed conditions in the DTPpop in bin 8.06 collocated with QTL detected for grain yield in previous studies under well-watered [14, 26, 52], drought stressed [52] and nitrogen deficient conditions [58]. A potential candidate gene is sucrose phosphate synthase (*sps1*) as suggested previously [26]. *Sps1* regulates sucrose synthesis and determines the partitioning of sucrose for export and storage. PVE and LOD for this QTL are not as high as typically expected for marker assisted selection but the presence across multiple environments and populations make this chromosomal segment highly interesting for further investigation.

### Flowering time QTL in bin 2.06

Two strong flowering time QTL mapped to bin 2.06 in the DTPpop and the LPSpop under well-watered and drought stressed conditions respectively. A potential candidate gene underlying the observed collocations is restorer fertility 8 (*rf8*) discovered earlier [59]. As a nuclear restorer gene *rf8* is responsible for pollen fertility and potentially accounts for the identified QTL.

### Good early vigor combined with earlier flowering will improve yields under terminal drought stress

Using two linked BC_1_F_2:3_ backcross populations allows to introgress the trait increasing alleles from either donor (LPSC7F64; DTPWC9F104) into the elite inbred line (CML491) using marker assisted selection. Overall, allele origin for specific traits was not exclusive under either condition indicating that additive effects of alleles from both parents were complementing each other and increasing grain yield under well-watered and drought stressed conditions. Within the seven chromosomal segments (2.02, 2.05, 2.06, 2.07, 5.02, 5.04, 8.06) with potential utility (early vigor, EPP, short ASI, early flowering, stay green) for breeders, six beneficial alleles were derived from the donor parents while 12 alleles were derived from the recurrent parent. Vigor (NDVI/PHT) was generally increased by alleles derived from CML491, EPP was increased by alleles derived from both parents in both irrigation treatments, while senescence was reduced by the allele derived from the donor under well-watered conditions. At the same time flowering time was consistently delayed by the allele derived from CML491. Alleles with beneficial effects on grain yield (and traits associated with grain yield) being derived from both parents does not come as a surprise since the release of a CIMMYT maize line (CML) typically implies selection for beneficial agronomic traits, vigor, disease tolerance and a certain level of drought tolerance. At the same time the improved performance of LPSC7F64 and DTPWF104 under drought partially relies on drought escape as a result of earlier flowering [35] explaining the origin of alleles advancing flowering from the donor parent in both populations evaluated in this study. It is not clear to what extent these results were affected by the use of a BC_1_F_2:3_ population since the genome of entries included in the current study only consisted of 25% derived from the donor. On the one hand high marker density used in this study allowed us to identify lines having ‘genetic target areas’ while at the same time reducing linkage drag. On the other hand using two BC_1_F_2:3_ populations (25% donor genome only) with a limited amount of entries (LPSPop: 220; DTPpop: 175) would have a had reduced genetic and phenotypic variation compared to traditionally used mapping populations [28, 31] making it more difficult to detect QTL with less strong effects.

The key to improved drought tolerance while at the same time maintaining yield potential in germplasm developed/discovered here, is to combine alleles conferring early vigor from the recurrent parent and alleles from the donor conferring earlier flowering. Good early vigor would allow for a rapid establishment, weed suppression, a deep rooting system and resulting improved water homeostasis under drought stressed conditions. Earlier flowering would allow plants to partially escape drought under environmental conditions with terminal drought [7] whereas it is expected to reduce yield potential under well-watered conditions. Reductions in yield potential could be avoided if selection for earlier flowering went along with a good early vigor [19]. Improved stay green would help mitigate detrimental effects of drought on plant performance.

### Identification of breeder ready germplasm

An advantage using BC_1_F_2:3_ backcross populations is that germplasm generated is likely to outperform parents and potentially current check hybrids [36]. Best offspring can immediately be incorporated into a breeding program as it is expected to combine favorable agronomic alleles from the recurrent parent and drought tolerance from the donor. Accordingly the highest yielding ten entries for all population-by-irrigation treatment combination (except LPSpop WW) used in this study outyielded the best check (CML312/CML444) by 32.5% (DTPpop WW) to 60% (DTPpop DS). Moreover three entries (((CML491/DTPWC9F104)//CML491)B2/CML503; ((CML491/LPSC7F64)//CML491)B154/CML503; ((CML491/LPSC7F64)//CML491)B218/CML503) ranked within the top ten across irrigation treatments. Best performing entries identified here under drought can therefore be used as new trait donor using phenotypic and/or molecular selection (after marker validation). Upon validation with a broader set of testers and evaluation in multi-environmental trials entries performing best across irrigation treatments could be released as CIMMYT maize lines (CML) combining superior agronomic performance of CML491 and drought tolerance of LPS or/and DTP, respectively.
