# Correction: Identification of QTL for Early Vigor and Stay-Green Conferring Tolerance to Drought in Two Connected Advanced Backcross Populations in Tropical Maize (*Zea mays* L.)

**DOI:** 10.1371/journal.pone.0163400

**Published:** 2016-09-15

**Authors:** Samuel Trachsel, Dapeng Sun, Felix M. SanVicente, Hongjian Zheng, Edgar Antonio Suarez, Raman Babu, Xuecai Zhang

Dr. Gary N. Atlin is listed in the author byline as the fifth author, but he has requested to be removed from the author list as he does not meet our authorship criteria. The correct citation is: Trachsel S, Sun D, SanVicente FM, Zheng H, Suarez EA, Raman B, et al. (2016) Identification of QTL for Early Vigor and Stay-Green Conferring Tolerance to Drought in Two Connected Advanced Backcross Populations in Tropical Maize (*Zea mays* L.). PLoS ONE 11(3): e0149636. doi:10.1371/journal.pone.0149636
